# Ocular Symptoms in General Medicine

**Published:** 1903-08-22

**Authors:** 


### The Hospital.

A Journal of

The Medical Sciences and Hospital Administration.

Vol. XXXIV.—No. 882. AUGUST 22, 1903.

### Ocular Symptoms in General Medicine.

There is no more attractive or effective answer to the critics of specialism than the argument that many of the observations made in special practice become, more or less promptly, of practical value and service in the larger field of general clinical work. If knowledge which is peculiar and restricted to-day will to-morrow prove itself competent to aid in the solution of diagnostic and therapeutic problems having a widespread claim, there is abundant justification for the policy which has rendered such knowledge possible. In this respect it may be safely said that of all the departments of special practice there is none which has a more secure defence than that of ophthalmology. Unfortunately, the study of ophthalmology presents certain initial difficulties, and these are apt to deter the general observer from an endeavour to prosecute it. Hence it often happens that not only are abnormal conditions of the eye regarded as the peculiar province of the ophthalmic surgeon, but the state of the ocular apparatus is excluded from the practitioner's scheme of examination. The resulting loss in efficiency is considerable. And this is the more to be regretted in view of the consideration that it could be largely avoided by a very moderate expenditure of time and trouble. Necessarily, without the ophthalmoscope the fundus oculi must remain a *terra incognita*, but it is no very serious task to acquire facility in the use of this method of observation, and those who make the effort find a ready and abundant reward. To take but a single example, the diagnosis of tubercular meningitis. It is a familiar experience that the clinical presentation of this disease is very often far removed from the classical description of the text-books. A moderate degree of fever and some tendency to drowsiness in a slightly ailing child may be the only obvious facts, and these may easily be interpreted as the results of chill or digestive disturbance. Certainly they afford no sure ground for diagnosis, and in the light of numerous parallel experiences having a favourable issue it is not surprising to find that frequently they fail to excite suspicion. Hence come instances of gravely erroneous prognosis and of neglected opportunities for early treatment. Now it is beyond question that many of these might be avoided by ophthalmoscopic examination. Evidences of gradually increasing congestion of the optic discs or the presence of tubercles in the choroid—conditions readily appreciated when ophthalmoscopic examination is cultivated as a routine experience—are facts of very exact significance, and their detection in connection with such symptoms as the above alters of course the entire outlook. And what is true in this regard of tubercular meningitis is equally true of many other diagnostic difficulties.

Passing next to the question of errors of refraction, it may be admitted that to deal adequately with these often demands an amount of time inconsistent with the claims of a busy practice. Yet by habit, and some ingenuity in organisation, this demand may be much reduced, and in any event, the practitioner apt with his ophthalmoscope and accustomed to the use of the test types, will readily determine whether a recurring frontal headache or other discomfort has a possible explanation in the presence of astigmatism or other refractive defect. Even if circumstances make it desirable that the correction of such defects shall be undertaken by an expert, their detection is an advantage to both practitioner and patient.

Again, the muscular apparatus of the eyeball may be quoted as well worthy of attention in reference to the light it throws on the problems of general medicine. Here little or no instrumental aid is necessary. Yet not infrequently observations in this direction are not pursued because the eye is, as it were, ruled off as a special department of practice. The truth is that disturbances of the actions of the ocular muscles, if they are to be marked as the property of any one special province, belong rather to neurology than to ophthalmic surgery. But what is to be desired is that these functions shall be brought as a matter of routine examination under the scrutiny of all those whose practice compels a wide outlook on the processes of disease. The pulse is common property. The tongue is common property. It were well to place the condition of the pupils, and indeed the actions of the ocular muscles generally, on the same platform, and to make them an essential part of the general examination of the patient. To justify this suggestion were an easy task. The relationship of ocular paralysis, of nystagmus, of the movements of the pupil, to organic nervous disease; the possible disparity in the size of the pupils caused by an intrathoracic tumour; the significance of the evidences of a former iritis—these alone are sufficient to show that the facts of the visual apparatus penetrate in many directions, and that it is much to be desired that those whose work is not confined within any special department should accustom themselves to appreciate these facts.

